# The Report on Diphtheria Antitoxin

**Published:** 1896-04-11

**Authors:** 


### The Report on Diphtheria Antitoxin.

The long-looked-for report of the medical superintendents of the Metropolitan Asylums Board on the use of antitoxic serum in the treatment of diphtheria has at last appeared, and although it is clear that on many points it is capable of diverse readings, its authors are certainly to be congratulated on having presented their facts in an orderly and well-tabulated form, and having drawn their conclusions in a temperate and impartial manner.

It is stated that no change has taken place during the year in the local treatment of the cases, nor has there been any new factor in the treatment other than the injection of antitoxin, to which, therefore, any improvement in the results must be attributed. Separate tables are given for each hospital, and summaries for the whole, and they are so arranged as to show the number of cases and the number of deaths at each age period, and at each day of the disease at which the treatment was commenced, and this both in regard to the cases treated by antitoxin and those of the total number admitted.

It would, however, be obviously futile to institute any comparison between the cases in which the antitoxin was used and the whole mass of cases treated in any preceding year, since the selection of the cases in which this treatment was to be employed was based on their severity, the milder cases being in most instances left under ordinary treatment. We are, therefore, driven to limit ourselves to a comparison between the total results obtained in the pre-antitoxin year, 1894, with the total results obtained in 1895, while antitoxin was being used, although it is clear that by so doing any benefit effected by antitoxin will not declare itself so markedly as it otherwise might do, in consequence of the results being mixed up with those of a crowd of cases in which the treatment was not employed.

What we find, then, is this, that while the mortality among all the patients during 1894, before the use of antitoxin, was 29·6 per cent., that of all cases treated in 1895, both with antitoxin or without, was 22·5, the apparent gain being a lowering of the death-rate by 7·1 per cent.

A more careful investigation, however, of the cases admitted during these two years shows that the gain has been very unequally distributed, and that the treatment has appeared to be much more actively beneficial to those subjected to it at an early day of the disease than to those in whom the malady had had time to become deeply rooted in the system; for while the reduction of mortality was comparatively slight among those who did not come under treatment until the fifth day and after, the death-rate among those who were treated on the first day of the disease was brought down in 1895 to only a fraction over half that which prevailed in 1894.

Implication of the larynx is one of the most serious complications of diphtheria, and if we take its occurrence as indicating a certain severity of disease, or, at any rate, as indicating a type of the disease which is fairly comparable with a similar type occurring in previous years, we find that the mortality of such cases fell from 62 per cent. in 1894 to 42·3 per cent. in 1895, and that not only was the percentage of cases in which tracheotomy was found necessary also reduced, but that the success of the operation was very much greater in the anti-toxin cases than it had been in the previous year when anti-toxin was not used, the percentage of mortality having fallen from 70·4 to 49·4.

Even still more startling results seem to have been achieved at the Winchmore Hill Hospital for Convalescents, where the diphtheria mortality during the three preceding years had stood at the alarming figure of 63 per cent. During 1895, however, the death-rate fell to 3·3 per cent., and if certain necessary deductions be made there still remained 53 cases of at least average severity, with only four deaths, a percentage of 7·5. This is a remarkable result.

It seems to us that the Asylums Board have been fully justified by results in the action they took in putting at the disposal of the medical superintendents of their hospitals a reliable and uniform antitoxic serum; and although we by no means shut our eyes to the complications which seem in a considerable number of cases to have followed the injections, we think that practitioners will be well advised to make such arrangements as to enable them to have a supply of reliable serum available in every town for immediate use, for if there is one fact more striking than another in this report it is the great advantage of the early use of the remedy.

